# Complicated Meckel's diverticulum concealed by massive mucinous ovarian carcinoma – A case report of the cautionary tale of surgical tunnel vision

**DOI:** 10.1016/j.ijscr.2025.111467

**Published:** 2025-05-26

**Authors:** Samith Minu Alwis, Georgi Atanasov, Marcos Vinicius Perini

**Affiliations:** Department of Surgery (Austin Health), The University of Melbourne, Melbourne, Victoria, Australia

**Keywords:** Cognitive bias, Meckel's diverticulum, Ovarian neoplasm, Surgical oncology, Case report

## Abstract

**Introduction:**

Cognitive bias, especially tunnel vision, in the clinical setting can predispose to catastrophic outcomes termed “never events”.

**Case presentation:**

A 58-year-old woman presented with abdominal pain on the background of chronic abdominal distension. Imaging indicated a massive ovarian lesion with presumed small bowel obstruction (SBO) secondary to mass effect. She underwent resection and staging workup. In addition to the 26.5 kg mucinous ovarian adenocarcinoma (MOC), she was intraoperatively found to have a Meckel's diverticulum (MD) with a band adhesion to proximal bowel (as the true precipitant of her SBO) and a contained enteric perforation, necessitating bowel resection and re-anastomosis. She made an excellent postoperative recovery and remains recurrence-free.

**Clinical discussion:**

In contrast to Meckel's diverticula which pose a preoperative diagnostic challenge, the massive MOC represented a distracting synchronous pathology which predisposed to tunnel vision. Surgical tunnel vision increases the risk of never events. A variety of systematic debiasing strategies have been proposed to raise awareness of cognitive bias but further research is still necessary to investigate the long-term clinical benefit of these strategies.

**Conclusion:**

Clinicians can readily incorporate debiasing techniques to raise awareness of unconscious biases, particularly the natural tendency to tunnel vision with glaring clinical findings. However, further study is required to explore the benefits of implementing debiasing techniques in the perioperative setting.

## Highlights

• Glaring clinical findings predispose to tunnel vision and consequent never events.
• Systematic debiasing strategies raise awareness of never events.
• Further study is needed into the value of debiasing strategies in clinical practice.

## 1 Introduction

Cognitive biases exist subconsciously in everyday clinical practice and can predispose to medical error. In surgery, these can yield deleterious consequences for patients termed “never events” which include inappropriate harm through unnecessary intervention or missed fatal diagnoses [1,2].

Tunnel vision, associated with anchoring and confirmation biases, has been linked to the occurrence of such never events. We present a rare case of a complicated Meckel's diverticulum (MD) concealed by a massive mucinous ovarian carcinoma (MOC) and identified only during operative management, highlighting the subconscious tendency to tunnel vision particularly in the setting of prominent radiological findings.

This case report adheres to the SCARE criteria [3].

## 2 Presentation of case

A 58-year-old female self-presented to the emergency department with a three-day history of severe generalised abdominal pain and vomiting with a year of progressive abdominal distension.

She reported three months of diffuse abdominal discomfort and poor oral intake with substantial weight loss. She had no infective symptoms, abnormal urinary symptoms, nor abnormal vaginal discharge. She was a non-smoker and non-drinker with asthma and a prior stroke, but no history of cancer, gastrointestinal or genitourinary disease. She did not have any regular pre-admission medications.

On examination, she was cachectic, haemodynamically stable and afebrile. She had a grossly distended, tense but non-peritonitic abdomen with diffuse mild tenderness.

**Fig. 1:**
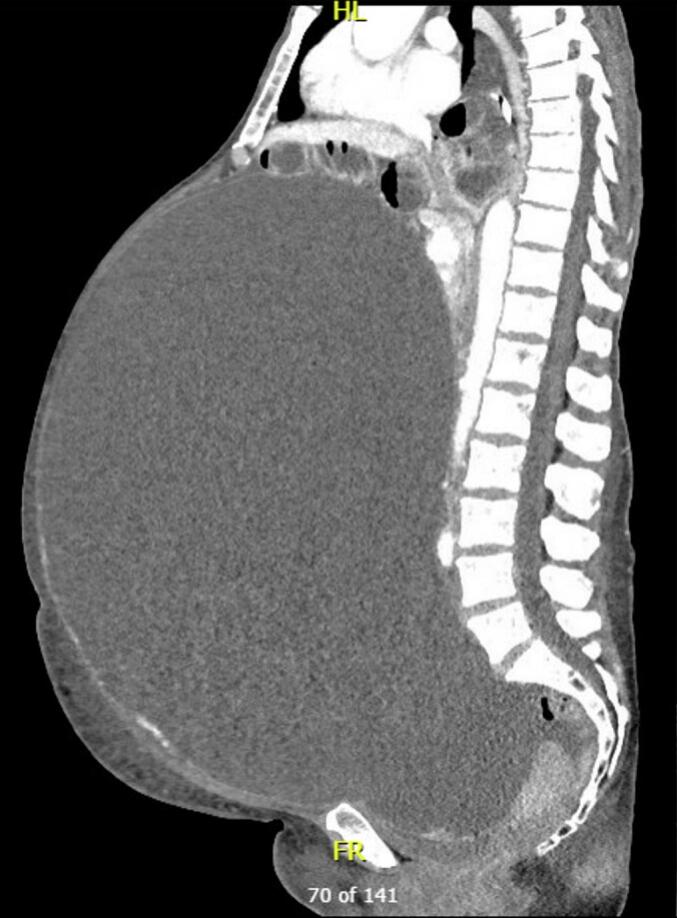
CT depicting a massive right ovarian lesion with displacement of bowel.

Blood tests demonstrated a leukocytosis (18 × 10^9^/L) and mildly elevated C-reactive protein [CRP] (12 mg/L). Her baseline tests were otherwise pristine with normal haemoglobin, kidney and liver function tests. Her cancer biomarkers were unremarkable except for a mildly elevated CA-125 (54 units/mL). Computed tomography (CT) demonstrated a right ovarian mass with internal mixed solid and cystic papillary appearance (Fig. 1). Marked mass effect was exerted on surrounding organs including the small intestine suggestive of small bowel obstruction (SBO). No clear transition point was seen (Video 1). A staging FDG-Positron Emission Tomography (PET) scan demonstrated activity confined to the right ovary.

A working diagnosis of acute-on-chronic SBO was established. This was presumed secondary to extrinsic compression with evidence of intra-lesional heterogeneity on CT and FDG-PET avidity raising suspicion for an ovarian malignancy. A virgin abdomen coupled with glaring imaging findings yielded a lower pre-operative probability of common causes and complications such as adhesions, closed loop SBO and ischaemic or perforated bowel.

Given the chronicity of the mass and logistics surrounding intraoperative involvement from the consulting gynaecology service, operative management was electively delayed. She instead underwent a workup for the resection of the ovarian mass. Supportive measures instituted included nasogastric decompression, analgesia optimisation, nausea management, and parental nutrition. During this period, while pain improved partially with analgesia she demonstrated ongoing nasogastric output but had normal vital signs, a non-peritonitic abdomen and unremarkable electrolytes with improved leukocytes and mildly elevated CRP).

**Fig. 2:**
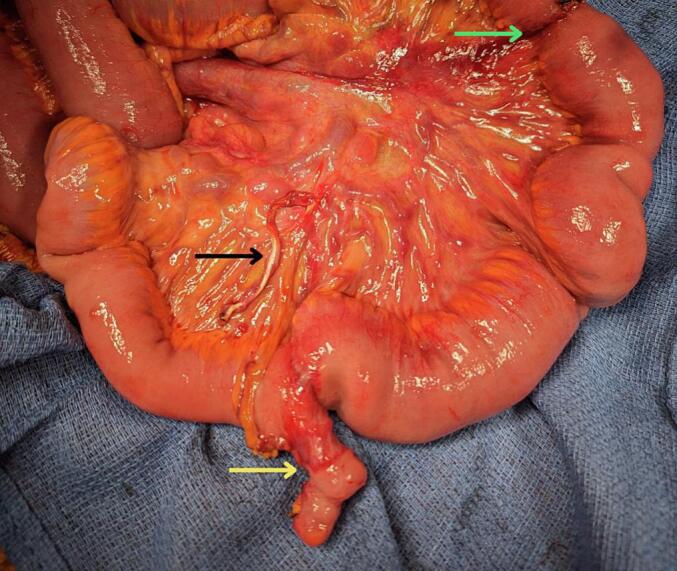
Meckel's diverticulum (yellow) complicated by band adhesion (black) with perforation at the site of adhesion (green). (For interpretation of the references to colour in this figure legend, the reader is referred to the web version of this article.)

Following preoperative optimisation and consensus in multidisciplinary discussions, a laparotomy (seven days after presentation) was performed with general surgical and gynaecology involvement. A large right ovarian cystic lesion weighing 26.5 kg was resected (Video 2). After the whole specimen was removed intact, intra-abdominal exploration identified a band adhesion (Fig. 2) between an incidentally identified MD and the proximal small intestine. Upon unravelling the bowel, a contained enteric perforation was evident 30 cm proximal to the MD where the band was compressing the small intestine (Video 3). Following *en bloc* resection of the large ovarian mass, bilateral salpingectomy, appendicectomy and omentectomy, a 40 cm enterectomy with entero-enteric anastomosis was performed to remove the MD and the site of perforation.

Histology revealed a MD and a grade 2 right MOC with differentials including primary MOC, or metastatic disease from a gastrointestinal (Krukenberg tumour) or pancreaticobiliary primary. The omentum, appendix, left ovary and fallopian tube were negative for malignancy. This was consistent with International Federation of Gynaecology and Obstetrics stage IB ovarian cancer.

Postoperatively, she was admitted to the intensive care unit. Her admission was complicated by intra-abdominal sepsis secondary to an anastomotic leak requiring further enterectomy and anastomotic revision. She also required drainage of an intra-abdominal simple fluid-containing collection. Despite these complications, she made an excellent recovery and was discharged home.

She underwent an interval FDG-PET one month postoperatively to assess for occult disease which may have been overshadowed on initial imaging in the setting of the ovarian mass. No residual PET-avid disease was found. She also underwent a completion gastroscopy and colonoscopy, both of which were unremarkable for any gastrointestinal lesions, increasing the likelihood of a primary ovarian neoplasm.

On gynae-oncology review, no adjuvant chemotherapy was recommended in the absence of extraovarian disease. Ten months postoperatively, she had a repeat PET scan negative for residual or recurrent disease, was progressively recovering from malnutrition and had begun returning to pre-morbid level of function.

## 3 Discussion

This is a unique diagnostic dilemma of SBO with synchronous rare pathology: primarily, a meso-diverticular band arising from a MD as the true precipitant, and secondarily, a distracting large MOC.

Primary MOC is a rare epithelial tumour accounting for <3 % of ovarian cancers and raises concern for a metastasis from breast or gastrointestinal primary [4]. Symptoms are often non-specific and include abdominal pain, bloating, distension and symptoms of mass effect. Our patient presented with such symptomatology and underwent routine workup and operative management from an oncological perspective as guided by our consulting gynaecology service.

While MD is the most common congenital gastrointestinal tract malformation, it has a similarly rare prevalence of 0.6–4 % [5]. It represents a remnant of the normally obliterated vitelline duct, presenting as a true ileal diverticulum. Contrary to our case, epidemiological risk factors for symptomatic MD include male sex and age < 50y [6]. Although bleeding is the most common presentation, MD can become complicated with perforation and most commonly, acute SBO (14–53 %). On standard CT, MD poses a diagnostic dilemma due to the inability to accurately distinguish it from non-pathological small intestine although a blind-ending pouch may be visible. Imaging can however more reliably identify complicated MD through radiological evidence of bleeding or complications [7]. In this case, unfavourable clinical features for MD coupled with a more salient cause for SBO did not justify targeted investigations such as CT enterography or nuclear imaging with technetium-99 m pertechnetate scintigraphy (diagnostic gold standard) [8]. Notably, an actively inflamed MD can be visible on FDG-PET though this was not present in our case – possibly overshadowed by the ovarian mass.

### 3.1 Tunnel vision in surgery

Our patient's presentation highlights the risks of cognitive bias in surgery, especially the natural tendency for tunnel visioning in everyday practice. Relevant biases encountered included anchoring, confirmation and search satisfaction biases with diagnostic momentum leading to early exclusion of alternative causes [9]. Preoperatively, the patient was responsive to analgesia and had reassuring vital signs and clinical examination which reduced the suspicion of sinister conditions such as perforation or ischaemic bowel. It could be argued that the preoperative assessment was consistent with *lex parsimoniae* and in the hypothetical absence of the abdominal mass, our patient may have proceeded to further evaluation and diagnosis of a MD. However, it is undoubtable that the presumed link between the mass and SBO expedited the intraoperative diagnosis of complicated MD.

Bias in surgery can yield catastrophic consequences including missed fatal diagnoses or inappropriate and harmful intervention. Anchoring and confirmation biases especially have been linked to surgical “never events” (e.g. wrong patient, wrong surgery) [1]. The literature recommends “debiasing” strategies that can be readily incorporated into clinical and radiological assessment. Clinicians can seek to avoid anchoring and diagnostic momentum by maintaining broad differentials through guided reflection and practicing multidisciplinary deliberation to challenge a working diagnosis asking, *“What else could this be?”* Confirmation bias can be mitigated through the use of open-ended questions and decision aids such as validated risk or pre-test probability calculators (e.g. Alvarado score for acute appendicitis) [10]. In multidisciplinary meetings, strategies can also include the delegation of a “devil's advocate” [11]. Search satisfaction can be mitigated by serial systematic re-assessment (alike to tertiary reviews in trauma) when key findings are identified. This can include the routine use of checklists to ensure a complete assessment. However, while all these strategies raise awareness of bias, implementation is hindered by time constraints with clinical workload, sleep deprivation and fatigue, and cognitive or physical multitasking leading to inattentiveness [12]. Importantly, there is also limited literature investigating debiasing strategies with no conclusive evidence to demonstrate any long-term clinical benefit [1,2]. This therefore warrants further investigation into biases and correlation with outcomes (especially incidence of complications and never events) particularly in the perioperative setting where there is significant risk of iatrogenic harm.

In our case, no clear transition point was reported on imaging and multidisciplinary discussion did not raise an alternative cause for her SBO. Misdiagnoses can therefore still occur (e.g. due to limitations of clinical or radiological assessment) highlighting the equal importance of retrospective multidisciplinary audit in evaluating decision-making and experimenting methods to identify and reduce the influence of tunnel vision (e.g. secondary independent reviews or second opinions). While our patient experienced a fortunate perioperative course in managing both pathologies, this case serves as a cautionary tale of the perils of surgical tunnel vision.

## 4 Conclusion

A natural tendency to tunnel vision exists in the setting of glaring clinical or radiological findings. Clinicians can readily incorporate debiasing strategies to raise their awareness of unconscious biases, but further study is needed investigate their clinical benefits in the perioperative setting.

The following are the supplementary data related to this article.

Video 1 CT scan depicting a massive right ovarian lesion (white) with displacement of intra-abdominal viscera and prominent fluid-filled, congested bowel loops (orange) with herniation into the mediastinum (red). Video 1

Video 2 Resection and removal of 26.5 kg mucinous ovarian carcinoma. Video 2

Video 3 Contained enteric perforation with evidence of torsion and chronic stricture secondary to mesodiverticular band adhesion. Video 3

## Consent

The patient was consented for the procedure and for presentation and publication of this manuscript.

## Ethical approval

Case study exempted from ethical approval at our institution.

## Funding

This research did not receive any specific grant from funding agencies in the public, commercial, or not-for profit sectors.

## Author contribution

SMA: Visualisation, Writing – original draft and review/editing.

GA: Data curation, Visualisation, Writing – original draft and review/editing.

MVP: Supervision, Conceptualisation, Data curation, Visualisation, Writing – original draft and review/editing, Guarantor.

## Guarantor

Marcos Vinicius Perini (MVP).

## Research registration number

Not applicable – Not a “First in Man” case report.

## Conflict of interest statement

The authors have no conflicts of interest to disclose.
